# Augmented Hemodynamic Responses in Obese Young Men during Dynamic Exercise: Role of the Muscle Metaboreflex

**DOI:** 10.3390/ijerph17197321

**Published:** 2020-10-07

**Authors:** Byung-Sun Lee, Kyung-Ae Kim, Jong-Kyung Kim, Hosung Nho

**Affiliations:** 1 Graduate School of Physical Education, Kyung Hee University, Seocheon-dong, Giheung-gu, Yongin-si, Gyeonggi-do 17104, Korea; shotace@khu.ac.kr; 2 Wellness IT Company Limited, 38, Teheran-ro 19-gil, Gangnam-gu, Seoul 06131, Korea; ggongae82@gmail.com (K.-A.K.); kyung19692002@yahoo.com (J.-K.K.)

**Keywords:** obese young men, hemodynamic response, dynamic exercise, post-exercise muscular ischemia, total vascular conductance

## Abstract

Studies found that cardiovascular responses to exercise are enhanced in individuals with obesity and are associated with a greater cardiac output (CO) response compared to normal weight controls. However, the mechanisms underlying these altered responses during dynamic exercise are not clear. We investigated whether the cardiovascular responses mediated by the muscle metaboreflex (MMR) activation are augmented in obese men during both static and dynamic exercise. Twenty males (10 obese (OG) and 10 non-obese (NOG)) were studied. Changes in CO, mean arterial pressure (MAP), and total vascular conductance (TVC) were compared between the two groups during dynamic handgrip exercise (DHE), post-exercise muscular ischemia (PEMI), and dynamic exercise corresponding to 40%, 60% and 80% workloads. Subjects completed 2 min of DHE at 30% of MVC, followed by 2 min of PEMI. MAP, CO, and TVC responses to DHE and dynamic exercise were significantly higher in OG, whereas there were no differences during PEMI. Increases in CO and MAP during mild to heavy dynamic exercise were seen in both groups, but the changes in these variables were greater in the OG. There were no significant differences in TVC between the two groups. Compared to NOG, the augmented blood pressure response to DHE and dynamic exercise in OG was associated with a greater increase in CO. Thus, the augmented CO and MAP responses were not associated with the activation of the MMR. Consequently, additional factors specific to obesity, such as the mechanoreflex, may have been involved.

## 1. Introduction

As the prevalence of obesity increases dramatically worldwide [1,2,3], it is considered a major public health problem. Obesity is a major risk factor for the development of chronic diseases such as type 2 diabetes, hypertension, coronary artery disease, and heart failure [4,5]. Regular exercise has been shown to reduce body fat as well as protect against cardiovascular diseases associated with obesity [6,7,8]. On the other hand, obesity produces excessive increases in arterial blood pressure (BP) during exercise [9,10]. These augmented BP responses may increase the risk of cardiac events associated with exercise (e.g., stroke, acute myocardial infarction) [11,12,13]. Therefore, understanding the mechanism that produces the altered hemodynamic responses during acute bouts of exercise may be important to reduce the future risk of cardiovascular disease.

Obese individuals with high BP increase their risk of evoking cardiovascular events and type 2 diabetes [14]. BP can be reduced using lifestyle modifications and regular exercise. For instance, aerobic exercise has been shown to decrease BP in obese individuals [15]. Paradoxically, obesity enhances an exaggerated BP response to exercise [16] and limits exercise safety. However, the mechanisms leading to excessive increases in BP during exercise in obese individuals are largely unknown. Both static and dynamic exercise increases BP, cardiac output (CO), heart rate (HR), and ventricular contractility with a reduction in peripheral vascular resistance during only dynamic exercise [17,18]. These cardiovascular adjustments are regulated by two major neural mechanisms: efferent inputs from central command, a feedforward neural drive originating in the higher brain center [19,20], and afferent inputs via group III and group IV sensory fibers arising from the contracting skeletal muscles [21]. The neural mechanism arising from group III and group IV receptors within skeletal muscles has been named as the exercise pressor reflex (EPR) [21,22,23]. The reflex is evoked by the activation of the mechanical receptors that are primarily composed of group III muscle afferents that initiate at the onset of contraction. The metabolic receptors that are primarily composed of group IV muscle afferents initiate later with accumulated metabolites within active skeletal muscle [23,24]. These neural reflexes may be good candidates to investigate the mechanisms that underline the exaggerated EPR in obese individuals.

Obesity is characterized by sympathetic nerve hyperactivity in organs such as the kidneys and skeletal muscles at rest [25]. The increase in sympathetic outflow has been partially associated with the dysfunction of the arterial baroreflex and the increased insulin resistance that derive from obesity [26,27]. The hemodynamic responses to exercise are altered by excessive sympathetic nerve activation mediated by the EPR in obese individuals [10,28]. Accordingly, these findings imply that overactive BP responses to exercise may be induced by the dysfunction of EPR.

In this regard, a previous study has reported that muscle metaboreflex (MMR)-induced muscle sympathetic nerve activity (MSNA) was reduced in normotensive obese women compared to normotensive lean women. There were no significant differences in mean arterial pressure (MAP) responses between the two groups during the isometric handgrip exercise [29]. This study demonstrated that hemodynamic responses mediated by the MMR are not augmented and the muscle vasodilatory responses are not altered in obese women. In contrast, another study investigated that a whole-body vibration caused exaggerated BP responses in normotensive obese women and suggested that this excessive response may be attributed to a mechanoreflex disfunction [9]. The inconsistency in BP response induced by the MMR activation could be due to differences in muscle mass (i.e., handgrip exercise vs. whole body exercise). Thus, further studies need to reveal why the BP response is potentiated in obese individuals, since aerobic exercise training is more effective for weight reduction than resistance training [30,31]. The aim of this study was to determine whether obesity-induced heightened BP response was due to the MMR-mediated increase in CO or peripheral vasoconstriction in obese individuals during dynamic exercise. We hypothesized that 1) obesity would increase arterial blood pressure and the larger obesity-induced BP response would mainly depend on enhanced CO response, 2) the excessive CO response would be mediated by an overactive MMR, since the MMR-induced pressor response mainly occurs via an increase in CO [32].

## 2. Methods

### 2.1. Participants

A total of 20 men (10 non-obese (NOG) and 10 obese (OB)), ages 21–25, participated in this study. Subjects were recruited from Kyung Hee University using flyers as advertisements. Only male subjects were selected because a previous study reported that resting and exercising blood pressure fluctuates during phases of the menstrual cycle [33]. Consequently, the effects of obesity on resting, exercising BP, and MMR will likely be less reliable if the phase of the menstrual cycle is not controlled. For the inclusion criteria, body mass index (BMI), a measurement commonly used to classify overweight and obesity in adults, was used. Subjects who met a >BMI of 30 kg/m^2^ (OB) and a <BMI of 25 kg/m^2^ (NOG) were qualified for this study. Individuals on medications that could affect cardiovascular function and BP responses were excluded from the study. All subjects were absent from any signs or symptoms of overt coronary heart disease based on a health history questionnaire and resting ECGs. The subjects were considered to be in good health. Subjects were instructed to refrain from alcohol, caffeine, and strenuous exercise 24 h prior to the study. This study was conducted only after review and approval by the Institutional Review Board (KHU 2014-G21) at Kyung Hee University Human Investigation Committee. The purpose and risks of the protocol were explained to each subject prior to giving written informed consent.

### 2.2. Experimental Design

Resting BP was measured in the brachial artery at the level of the heart while each subject was in a seated position. After 5 min of rest, at least two measurements were collected, 5 min apart, using a sphygmomanometer. For the baseline BP, the average of the two values was used. To determine the relative exercise intensities of the three workloads used in this study (40%, 60% and 80% VO_2peak_), a maximal exercise test was performed on a cycle ergometer (Monark 828, Sweden). The protocol began with 2 min of unloaded cycling, followed by increases of 30 watts/min, until the subjects could no longer maintain a cadence of 60 rpm. Breath-by-breath pulmonary gas exchange data was collected continuously with an Ultima CPX Metabolic Measurement Cart (Medgraphic, St. Paul, Minnesota, USA). The VO_2peak_ (peak oxygen consumption) obtained from this test was used as an index of functional capacity.

To perform dynamic handgrip exercises, the maximal voluntary contraction (MVC) of the dominant forearm was determined by having the subjects squeeze a hand grip dynamometer three times at maximal effort. This test was used as an index of maximal muscle strength. The highest value was used to determine the relative work intensity during the experimental protocol. Following a 10-min rest period in a chair, subjects performed a dynamic handgrip exercise for 3 min at an intensity of 30% of MVC consisting of a rhythmic (30 compressions/min paced by a metronome) handgrip. To provide visual feedback to the subjects and allow them to maintain the designated work intensity, the target force was displayed on the handgrip device and was used to confirm that the prescribed relative exercise intensity was achieved in each subject. To induce skeletal muscle metaboreflex, the 3 min dynamic handgrip exercise was followed immediately by 2 min of circulatory occlusion induced by inflation of a blood pressure cuff (placed around the non-dominant upper arm) to suprasystolic levels (~200 mmHg) [34,35]. This post-exercise muscular ischemia (PEMI) was used only to isolate the cardiovascular effects of the metaboreflex from those of both the central command and the muscle mechanoreflex.

**Figure 1:**
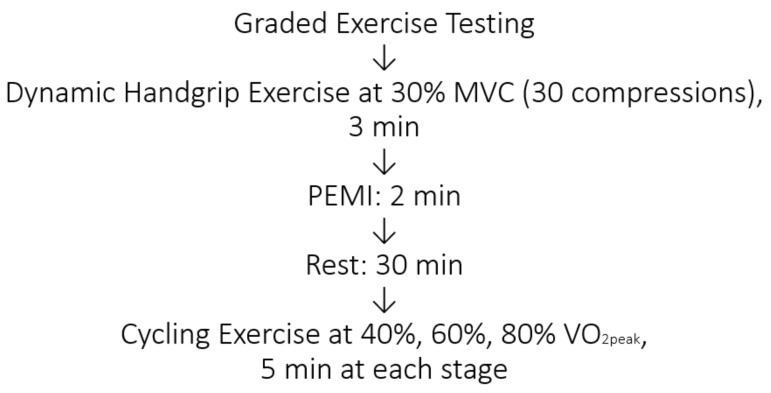
Experimental design.

Following at least 30 min, subjects then completed 3 bouts of dynamic cycling exercises at constant submaximal workloads corresponding to 40%, 60% and 80% of their predetermined VO_2peak_ values. The duration of each workload were 3–5 min (Figure 1). Subjects performed these submaximal trials on the same day without a rest period between the three workloads. The workload was achieved when they reached a steady state.

#### 2.2.1. Measurements of Hemodynamic Variable

Impedance cardiography (Physioflow, Manatec Biomedical, Poissy, France) was used to continuously measure stroke volume (SV) and heart rate (HR) from rest throughout sessions of metaboreflex activation and cycling exercise. The device allows for the calculation of real-time cardiac output (CO) in healthy subjects [36,37,38]. The Physioflow has been previously validated and is highly correlated with the direct Fick method at rest and during submaximal and maximal exercise [37,39]. The impedance method has been previously employed and described in detail in similar experimental settings published by our laboratory [33,40,41]. Briefly, the bioimpedance device consisted of two impedance cardiography electrodes placed above the supraclavicular fossa at the base of the left side of the neck, two electrocardiography electrodes used for recording the ECG, and two electrodes placed at the xiphoid process. The Physioflow measured the change in transthoracic impedance during the cardiac cycle (Bougault et al. 2005). CO was calculated according to the following formula: CO = HR × Svi × BSA, where HR is measured from the R-R interval determined from the first derivative of the ECG. SVi is the SV index (i.e., SV/body surface area (BSA)). Body surface area (BSA; meters squared) was determined according to the Haycock formula: BSA = 0.024265 × BM0.5378 × H0.3964, where BM is body mass in kilograms and H is height in centimeters.

Exercising systolic (SBP) and diastolic (DBP) blood pressure were measured in the brachial artery at the level of the heart by a standard manual sphygmomanometer in steady-state conditions during cycling exercise. The same sphygmomanometer was used for SBP and DBP assessments, which were performed in the nondominant arm by the same investigator throughout all protocol sessions during PEMI. MAP was calculated using the formula: MAP = ((SBP − DBP) × 1/3) + DBP. Total vascular conductance (TVC) is an index of peripheral vasoconstriction and was calculated as Q/MAP.

#### 2.2.2. Cold Pressor Response (CPT)

The cold presser test is a cardiovascular test performed to determine the BP response to a nonexercised selective sympathoexcitory stimulus [35]. Ten min after the collection of baseline data in a chair, this procedure was conducted by immersing their dominant hand in ice water (7 °C) to the level of the wrist for 1 min. Systolic blood pressure (SBP) and diastolic blood pressure (DBP) were measured in the non-dominant arm during this period.

### 2.3. Statistical Analysis

Hemodynamic data collected by means of impedance cardiography during the metaboreflex test and cycling exercise were averaged over the last 30 s periods in the steady-state condition. Mean values of HR, SV, CO, and TVC for each 30 s interval at rest and during exercise were used for comparison purposes. Blood pressure was measured between the 4th and 5th min of each workload during cycling exercises and at the last 30 s during PEMI. To compare the effects of obesity over workloads and between groups, two way repeated-measures ANOVA (SigmaStat 4.0, Systat Software, Inc, Tulsa, OK, USA) and Tukey’s post-hoc test were used. Mean values of all variables were compared between groups via an independent t test. Statistical significance was accepted at *p* < 0.05. Values are expressed as mean ± Standard error. In order to detect a statistically significant change (*p* < 0.05) of 10% in SBP between two groups, a power test (power = 0.80) indicated that each group needed 10 subjects. Effect size (ES) was determined using Cohen’s D formula as the mean difference divided by the pooled standard deviation.

## 3. Results

Subject characteristics are summarized in Table 1. The subjects in the NOG and OG were age matched. Both resting SBP and DBP were significantly higher in the OG compared to the NOG. There were significant differences between the two groups in the level of body mass indices (BMI) and cardiorespiratory fitness (i.e., VO_2peak_).

**Figure 2:**
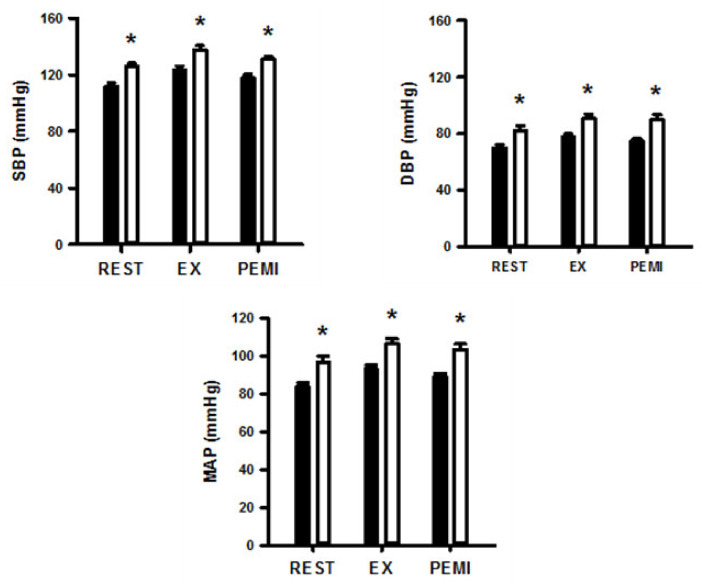
Systolic blood pressure (SBP), diastolic blood pressure (DBP), and mean arterial pressure (MAP) during dynamic exercise and post-exercise muscular ischemia (PEMI) in normal and obese individuals. Black bar, normal; open bar, obese. * *p* < 0.05, vs. normal individuals.

**Figure 3:**
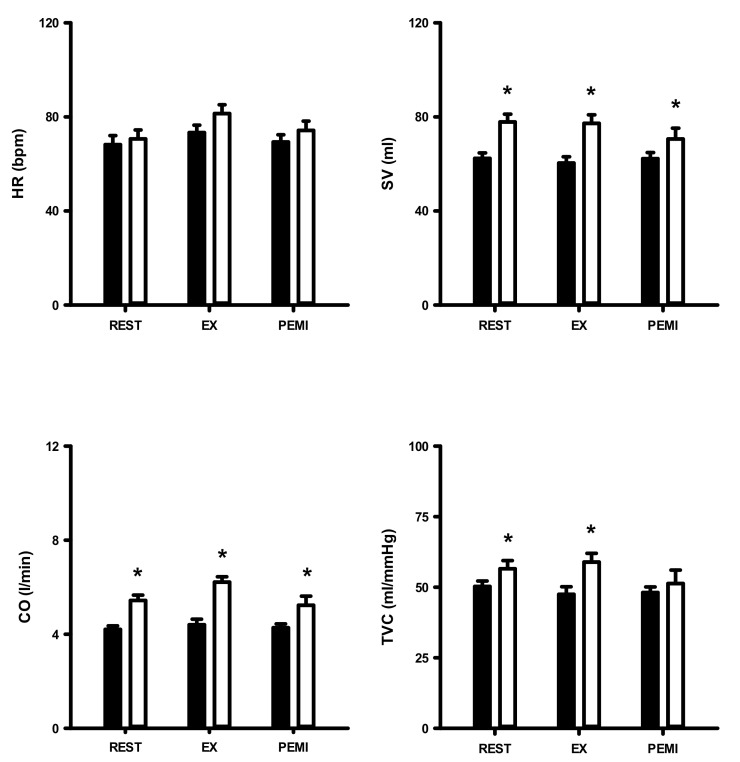
Heart rate (HR), stroke volume (SV), cardiac output (CO), and total vascular conductance (TVC) during dynamic exercise and post-exercise muscular ischemia (PEMI) in normal and obese individuals. Black bar, normal; open bar, obese. * *p* < 0.05, vs. normal individuals.

**Figure 4:**
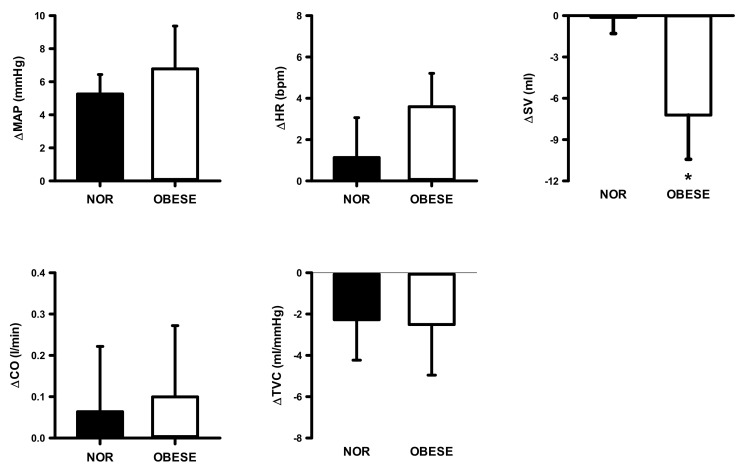
Changes from rest in mean arterial pressure (MAP), heart rate (HR), stroke volume (SV), cardiac output (CO), and total vascular conductance (TVC) during post-exercise muscular ischemia (PEMI) in normal and obese individuals. Black bar, normal; open bar, obese. * *p* < 0.05, vs. normal individuals.

Figure 2 shows SBP, DBP, and MAP values at rest and in response to 3 min of static exercise and 2 min of muscle metaboreflex activation in both groups. There were significantly higher SBP, DBP, and MAP in obese group at rest and during static exercise (ES = 1.72, ES = 1.64, and ES = 1.78, respectively) and muscle metaboreflex activation (ES = 2.15, ES = 1.79 and ES = 1.98, respectively). Figure 3 shows HR, SV, CO, and TVC at rest and in response to 3 min of static exercise and 2 min of muscle metaboreflex activation in both groups. There were significant increases in CO, SV and TVC at rest and during static exercise in the OG compared with NOG whereas no significant difference in HR occurred. The MMR significantly increased CO in OG compared with the NOG. There was no difference in TVC between the two groups. Absolute changes in the hemodynamic data from rest in response to 2 min of metaboreflex activation are shown in Figure 4. There were no significant differences in MAP, HR, SV, CO, and TVC in both conditions.

**Figure 5:**
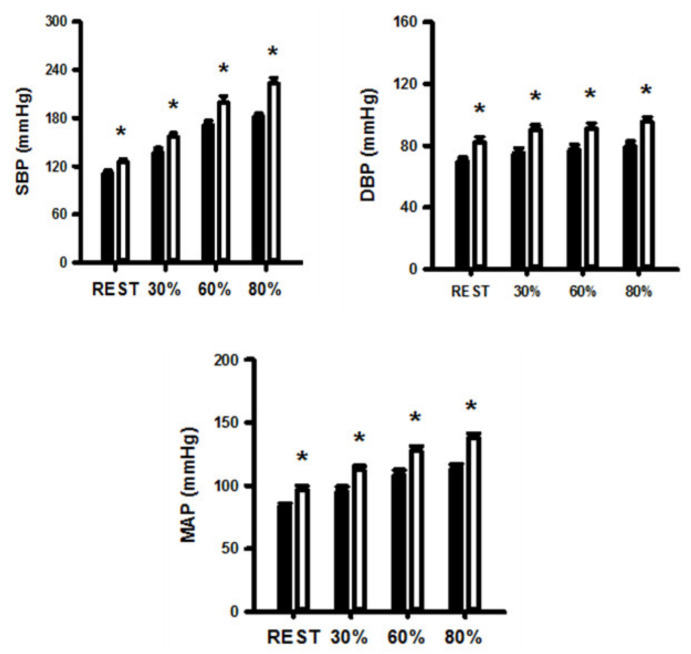
Systolic blood pressure (SBP), diastolic blood pressure (DBP), and mean arterial pressure (MAP) at rest and during dynamic exercise in both normal and obese individuals. Black bar, normal; open bar, obese. * *p* < 0.05, vs. normal individuals.

Figure 5 shows SBP, DBP, and MAP at rest and during aerobic exercise in the OG and NOG. The SBP, DBP and MAP were significantly increased in both groups, but these variables were significantly higher in OG than NOG when exercise intensity increased from rest to heavy exercise.

**Figure 6:**
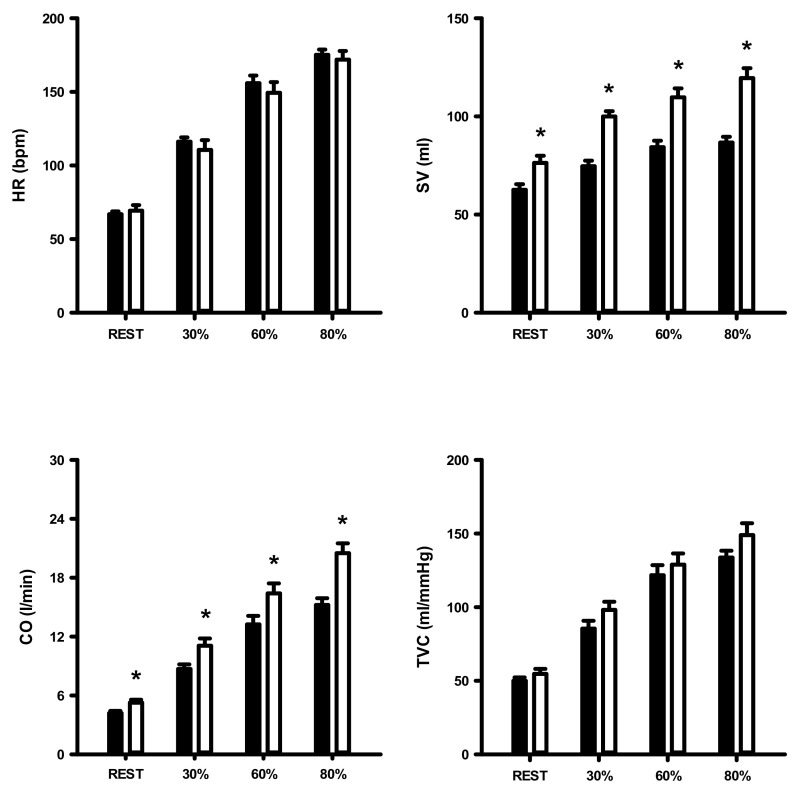
Heart rate (HR), stroke volume (SV), cardiac output (CO), and total vascular conductance (TVC) at rest and during dynamic exercise in both normal and obese individuals. Black bar, normal; open bar, obese. * *p* < 0.05, vs. normal individuals.

Figure 6 shows SBP, DBP and MAP at rest and during aerobic exercise in the OG and NOG. The SBP, DBP and MAP were significantly increased in both groups, but these variables were significantly higher in OG than NOG when exercise intensity increased from rest to heavy exercise. Figure 6 shows HR, SV, CO and TVC at rest and during aerobic exercise in both groups. There was a higher increase in SV and CO in OG compared with NOG during increasing workloads, whereas no significant differences in HR and TVC occurred.

**Figure 7:**
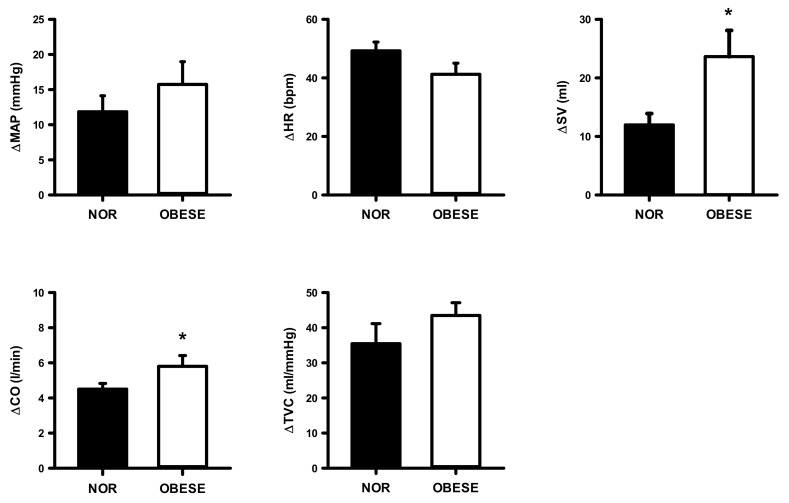
Changes from rest in mean arterial pressure (MAP), heart rate (HR), stroke volume (SV), cardiac output (CO), and total vascular conductance (TVC) during the 30% of VO_2peak_ workload in normal and obese individuals. Black bar, normal; open bar, obese. * *p* < 0.05, vs. normal individuals.

**Figure 8:**
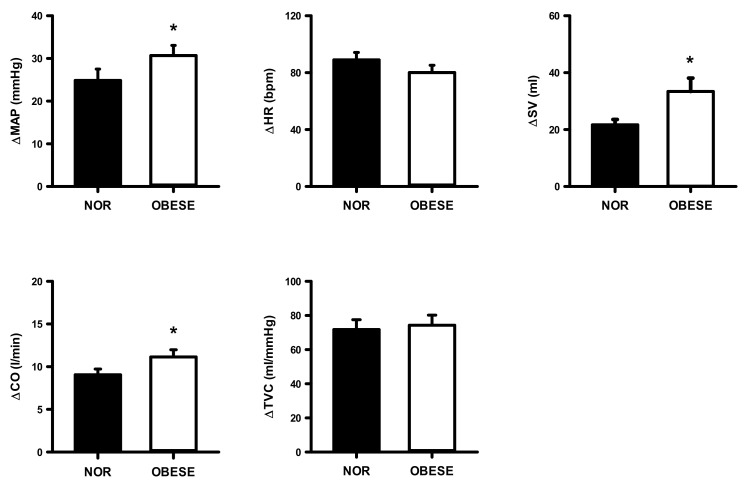
Changes from rest in mean arterial pressure (MAP), heart rate (HR), stroke volume (SV), cardiac output (CO), and total vascular conductance (TVC) during the 60% of VO_2peak_ workload in normal and obese individuals. Black bar, normal; open bar, obese. * *p* < 0.05, vs. normal individuals.

**Figure 9:**
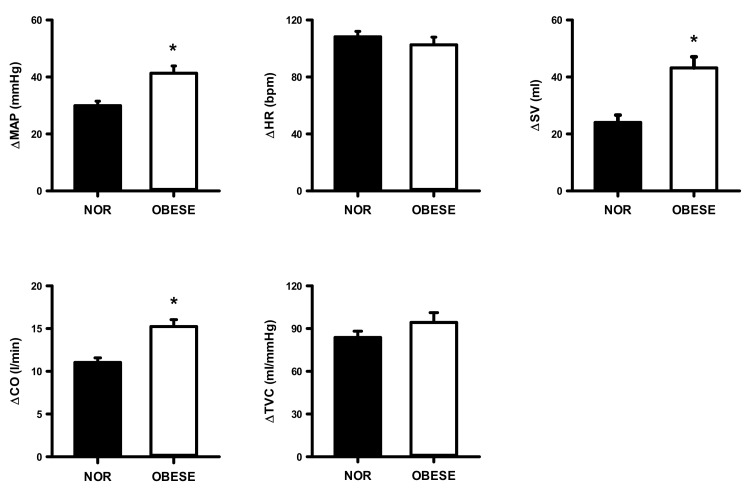
Changes from rest in mean arterial pressure (MAP), heart rate (HR), stroke volume (SV), cardiac output (CO), and total vascular conductance (TVC) during the 80% of VO_2peak_ workload in normal and obese individuals. Black bar, normal; open bar, obese. * *p* < 0.05, vs. normal individuals.

Figure 7, Figure 8 and Figure 9 show changes from rest in mean arterial pressure (MAP), HR, SV, CO and total TVC during the 40%, 60%, 80% of VO_2peak_ workload in both groups. The OG had a significantly higher increase in MAP and CO only at 60% (ES =0.83, ES = 1.96, respectively) and 80% (ES = 1.96, ES = 2.63, respectively) workloads compared with the NOG.

### Cold Pressor Test

The cold pressor test increased MAP from baseline in both groups. However, the magnitude of the increase was not significantly different between the obese group and non-obese group (OG: Δ15 ± 3 mmHg vs. NOG: Δ13 ± 2 mmHg).

## 4. Discussion

Our new findings show that SBP, DBP, MAP, SV and CO responses during the activation of central command, mechanoreceptors, and metaboreceptors induced by dynamic cycling exercise were augmented in obese young individuals compared with non-obese individuals. The same pattern was observed in both groups during a dynamic handgrip exercise. Although the absolute change in TVC was similar between groups with increasing workloads, overall CO was higher at rest and during exercise in OG compared with NOG. However, there was no difference in the MMR-mediated MAP and CO responses between both groups. Consequently, the current study demonstrated that obese individuals had an exaggerated blood pressure response to dynamic exercise and suggests that the mechanism behind this overactive response may occur due to a higher CO induced by other factors besides MMR.

### 4.1. Hemodynamic Responses to Exercise

An abnormal exercising BP response in normotensive individuals leads to a higher risk of future hypertension [42]. In fact, overweightness and obesity are associated with higher BP responses to dynamic exercise compared with normal weight counterparts [9,29,43,44]. Understanding these alterations in cardiovascular adjustments is important since they are associated with incidents of hypertension and an increased risk of cardiovascular disease [45,46]. Despite findings demonstrating that obesity alters cardiovascular hemodynamics during exercise, there has been a lack of evidence revealing the mechanism contributing to obesity-induced overactive BP response during dynamic exercise. Hence, we examined whether the excessive rise in BP initiated by exercise is primarily due to the MMR-mediated increase in CO and peripheral vasoconstriction in obese individuals. The present study found that the MAP was higher in OG at rest and during 40%, 60% and 80% exercise workloads compared to NOG. CO was significantly increased in both groups with increasing exercise workloads; however, the CO changes from baseline were greater in the obese group than those observed in NOG, with little change in peripheral vascular conductance. The same pattern occurred during the dynamic handgrip exercise. These observations indicate that in obesity, hypertensive BP responses to exercise are mainly due to an excessive increase in CO.

### 4.2. Effect of Obesity on the Metoboreflex-Mediated Pressor Response during Exercise

It is well documented that one of the neural controls of the cardiovascular adjustments during exercise is the EPR [19,20,21]. Previous studies have demonstrated that obesity induces a hypertensive response in circulatory hemodynamics and these alterations are associated with abnormalities of the EPR [9,29]. Dipla and colleague [9] examined the extent which obese women compared with lean women exhibited differences in MAP induced by involuntary contraction. This study found that involuntary contractions evoked substantially higher BP response in normotensive obese women than lean women. Another study showed that, in normotensive obese women, MSNA, BP and forearm vascular resistance were higher during static exercise compared with their lean normotensive counterparts whereas MSNA induced by the MMR activation was attenuated in obesity [29]. However, these previous studies were limited to the effect of static exercise or involuntary contraction to hemodynamic responses in normotensive middle-aged obese women. In our study, despite the fact that MAP and CO were significantly higher in OG during DHE and PEMI compared to NOG, there were no differences in the changes in CO by MMR activation between the two groups. With an increase in HR, this observation is offset by a fall in SV due to the high afterload induced by high BP [47]. In support of our findings, a previous study found that there were no differences in MAP, CO and peripheral vasoconstriction responses to the MMR activation between metabolically healthy obese individuals and non-obese individuals [48]. Taken together, these findings suggest that obesity evokes heightened BP by an increase in CO response to static or rhythmic exercise; yet this alteration is not associated with the MMR.

On the other hand, Milia and colleague [48] reported that obese individuals with metabolic syndrome (MS) had a greater BP response to the MMR activation due to peripheral resistance compared to the control and healthy obese groups. Similarly, another study demonstrated that weight loss reduced an increase in MAP by a fall in peripheral resistance to the MMR activation in individuals with high BMI (i.e., >40 of BMI) [49]. Findings indicate that in obese and MS condition, the MMR resulted in greater increase in systemic vascular resistance and the pressor response is mainly mediated via peripheral vasoconstriction. Thus, peripheral vasoconstriction may play an important role in mediating the MMR pressor response in obese and MS condition. Although there were no differences in TVC between the two groups after the MMR activation, our study is limited by the fact that we were not able to rule out the effect of MS on the MMR-induced peripheral vasoconstriction.

The current study extends previous studies by demonstrating that obesity enhanced an excessive BP response to dynamic cycling exercise, mainly via an increase in CO. Obesity had greater MAP and CO responses during cycling exercise compared to the NOG. There were also significant differences in the absolute changes in hemodynamic data in response to exercise between the two groups. The cycling exercise resulted in a substantial increase in SV, CO and MAP in the obesity condition. Thus, our findings suggest that the mechanism that alters hemodynamic responses in obese individuals during exercise may be induced by another component of the EPR.

The CPT is a simple and non- invasive test to assess central sympathetic activation. In our study, the branchial BP response to the CPT was used as an index of central sympathetic reactivity. Similar to a previous study [50], we did not observe any difference in the magnitude of this response between the NOG and OBG. Thus, it is assumed that an exaggerated BP response during exercise in the OBG correlates with EPR.

### 4.3. Effect of Obesity on the Mechanoreflex-Mediated CO Response during Exercise

There has been a lack of evidence suggesting that the central command or the mechanoreflex plays an important role in the exercise-induced overactive hemodynamic responses in individuals with obesity since it is difficult to isolate these reflexes in humans. In previous studies, involuntary contractions induced by a whole-body vibration or passive dynamic stretching of skeletal muscle have been used to only activate group III muscle afferents [9,51]. These investigators found that the mechanism mediating an increase in BP response initiated by the mechanoreflex activation was attributed to an increase in CO. Similarly, we observed in the OBG that the excessive BP response to exercise is mediated via increases in CO and SV across a broad range of workloads. These observations suggest that the muscle mechanoreflex may have a stronger control over the sympathetic activity to the heart and raises pressure by increases in CO during dynamic exercise. Thus, it appears that the major mechanism mediating an exaggerated BP response to exercise depends primarily on an increase in CO induced by this reflex activation in obese individuals. Further studies are needed to determine the role of the mechanoreflex in contributing to increases in CO and BP.

### 4.4. Limitations

We cannot address to what extent aerobic fitness can buffer the exercising BP response in the OG exactly. Kokkinos and colleague [12] demonstrated that low cardiorespiratory fitness is associated with a higher BP response during submaximal exercise, suggesting that increased fitness may attenuate this abnormal rise in BP. Our study cannot exclude the effect of low physical fitness on BP response during exercise, since OG had lower a VO_2peak_ compared to NOG. Another limitation is that the effect of MS is not considered to determine the BP response to MMR activation. MS shifts the mechanism of the MMR from mainly increases in CO to the peripheral vasoconstrictor. However, the role of MS was not investigated in the current study. Obese individuals are characterized by endothelial function, arterial stiffness, and overactive sympathetic activity [52,53,54], leading to cardiovascular alteration in response to exercise. Future studies need to reveal the role of these factors contributing to the high BP response to exercise.

### 4.5. Clinical Implications

The effect of obesity on the BP response to exercise has clinical implications. It is well known that exercise evokes a greater BP response in pathological conditions such as prehypertension and hypertension due to dysfunction of the EPR and leads to cardiovascular events (e.g., stroke/acute myocardial infarction) [34,55]. Although exercise training that involves aerobic exercise is recommended in order to lose body weight, it is important for obese individuals with high BP to monitor accentuated BP responses to exercise. An excessive BP response, even to low-intensity exercise, appears to be a simple noninvasive clinical test to determine if early treatment to prevent future hypertension is necessary.

## 5. Conclusions

In summary, this study investigated that the exercise-induced excessive BP response is mainly due to an increase in CO mediated by the MMR activation in OG. There was no difference in absolute changes in the MAP initiated by the MMR activation between the two groups. Despite the similar magnitude of the increases in peripheral vasodilation in both groups, the OG had higher BP responses due to an increase in CO during cycling exercise compared to NOG. Accordingly, the current study suggests that alterations in cardiovascular responses during exercise may not be attributable to overactive MMR in obese individuals.

## Figures and Tables

**Table 1:** Physical characteristics of subjects.

Variables	Normal Group (*n* = 10)	Obese Group (*n* = 10)
Age (years)	22.42 ± 1.30	23.71 ± 1.09
Height (cm)	175.10 ± 2.14	175.44 ± 2.27
Body weight (kg)	67.11 ± 2.43	95.35 ± 2.60
BMI (kg/m^2^)	21.82 ± 0.40	30.90 ± 0.61 *
SBP (mmHg)	111.32 ± 3.07	128.19 ± 2.43 *
DBP (mmHg)	73.29 ± 2.06	86.87 ± 2.67 *
HR (bpm)	66.33 ± 3.02	73.65 ± 4.88
VO_2peak_ (mL/kg/min)	44.60 ± 4.28 *	35.42 ± 2.10

Values are mean ± standard error. BMI, body mass index; SBP, systolic blood pressure; DBP, diastolic blood pressure; HR, heart rate; VO_2peak_, peak oxygen uptake. * *p* < 0.05 vs. normal group.
